# Piperazine-1,4-diium naphthalene-1,5-disulfonate

**DOI:** 10.1107/S1600536811038955

**Published:** 2011-09-30

**Authors:** Bin Wei

**Affiliations:** a Ordered Matter Science Research Center, Southeast University, Nanjing 211189, People’s Republic of China

## Abstract

The title molecular salt, C_4_H_12_N_2_^2+^·C_10_H_6_O_6_S_2_^2−^, consists of a piperazinium cation and a 1,5-naphthalene­disulfonate anion. Crystallographic inversion centers are situated at the center of the ring of the dication as well as at the midpoint of the central carbon–carbon bond in the dianion. In the crystal, inter­molecular N—H⋯O hydrogen bonds link the cations and anions.

## Related literature

The title compound was obtained during attempts to obtain dielectric-ferroelectric compounds. For general background to ferroelectric metal-organic frameworks, see: Wu *et al.* (2011 ▶); Ye *et al.* (2006 ▶); Zhang *et al.* (2008 ▶, 2010 ▶); Fu *et al.* (2009 ▶). 
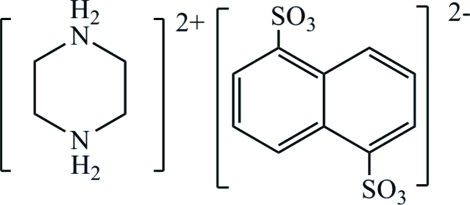


## Experimental

### 

#### Crystal data

C_4_H_12_N_2_^2+^·C_10_H_6_O_6_S_2_^2−^
*M* *_r_* = 374.42
Monoclinic, 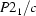

*a* = 11.997 (2) Å
*b* = 7.2959 (15) Å
*c* = 9.1453 (18) Å
β = 96.00 (3)°
*V* = 796.1 (3) Å^3^
*Z* = 2
Mo *K*α radiation
μ = 0.37 mm^−1^
*T* = 293 K
0.20 × 0.20 × 0.20 mm

#### Data collection

Rigaku SCXmini diffractometer
Absorption correction: multi-scan (*CrystalClear*; Rigaku, 2005 ▶) *T*_min_ = 0.955, *T*_max_ = 0.955
7956 measured reflections
1827 independent reflections
1629 reflections with *I* > 2σ(*I*)
*R*_int_ = 0.031

#### Refinement

*R*[*F* ^2^ > 2σ(*F* ^2^)] = 0.034
*wR*(*F* ^2^) = 0.088
*S* = 1.11
1827 reflections
109 parameters
H-atom parameters constrained
Δρ_max_ = 0.26 e Å^−3^
Δρ_min_ = −0.36 e Å^−3^

### 

Data collection: *CrystalClear* (Rigaku, 2005 ▶); cell refinement: *CrystalClear*; data reduction: *CrystalClear*; program(s) used to solve structure: *SHELXS97* (Sheldrick, 2008 ▶); program(s) used to refine structure: *SHELXL97* (Sheldrick, 2008 ▶); molecular graphics: *SHELXTL* (Sheldrick, 2008 ▶); software used to prepare material for publication: *SHELXTL*.

## Supplementary Material

Crystal structure: contains datablock(s) I, global. DOI: 10.1107/S1600536811038955/vm2121sup1.cif

Structure factors: contains datablock(s) I. DOI: 10.1107/S1600536811038955/vm2121Isup2.hkl

Supplementary material file. DOI: 10.1107/S1600536811038955/vm2121Isup3.cml

Additional supplementary materials:  crystallographic information; 3D view; checkCIF report

## Figures and Tables

**Table 1:** Hydrogen-bond geometry (Å, °)

*D*—H⋯*A*	*D*—H	H⋯*A*	*D*⋯*A*	*D*—H⋯*A*
N1—H1*A*⋯O2^i^	0.90	1.91	2.7357 (19)	153
N1—H1*B*⋯O3^ii^	0.90	1.91	2.7670 (19)	159

Symmetry codes: (i) 
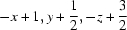
; (ii) 
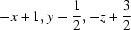
.

**Fig. 1.**
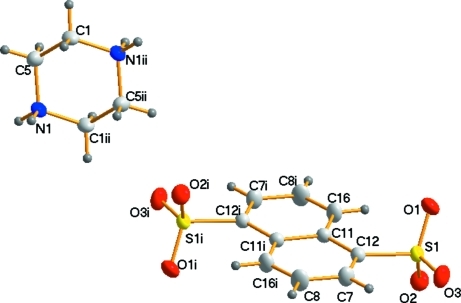
The molecular structure of the title compound with atomic numbering scheme. Displacement ellipsoids are drawn at the 30% probability level. Symmetry codes: (i) -x + 1, -y, -z + 2; (ii) -x + 2, -y, -z + 1.

**Fig. 2.**
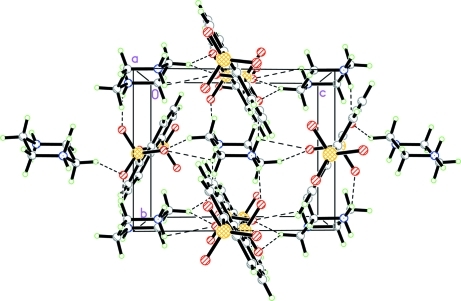
A view of the packing of the title compound along the a axis. Dashed lines indicate hydrogen bonds.

## supplementary crystallographic information

### Comment

Dielectric-ferroelectrics are an interesting class of materials, comprising organic ligands,metal-organic coordination compounds and organic-inorganic hybrids (Fu *et al.*, 2009; Zhang *et al.*, 2010; Zhang *et al.*, 2008; Ye *et al.*, 2006). Unfortunately, the dielectric constant of the title compound as a function of temperature indicates that the permittivity is basically temperature-independent. Below the melting point (402-403K) of the compound, we have found that the title compound has no dielectric disuniformity from 80 K to 405 K. Here we descibe the crystal structure of this compound.

The asymmetric unit of the title compound consists of a half piperazinium cation and a half 1,5-naphthalenedisulfonate anion (Fig. 1). The complete complete molecular structures are generated by inversion centers at the center of the piperazinium ring and at the midpoint of the central carbon-carbon bond in the naphthalene ring. The best planes through the piperazinium ring and the naphthalene ring make a dihedral angle of 80.96 (8)°. The cations and anions are connected by intermolecular N—H···O hydrogen bonds, which contribute to the stability of the crystal structure (Fig. 2 and Table 1).

### Experimental

The title compound was obtained by the addition of 1,5-naphthalenedisulfonate acid (3.62 g, 0.01 mol) to a solution of piperazine (0.88 g, 0.01 mol) in water, in the stoichiometric ratio 1: 1. Good quality single crystals were obtained by slow evaporation after two days (yield: 48%).

### Refinement

All H atoms were placed in geometrically idealized positions and constrained to ride on their parent atoms with C—H = 0.93 Å-0.97 Å, N—H = 0.90 Å and with *U*_iso_(H) = 1.2 *U*_iso_(C, O) or 1.5 *U*_iso_(C) for methyl H atoms.

### Crystal data

**Table:**

C_4_H_12_N_2_^2+^·C_10_H_6_O_6_S_2_^2^^−^	*Z* = 2
*M**_r_* = 374.42	*F*(000) = 392
Monoclinic, *P*2_1_/*c*	*D*_x_ = 1.562 Mg m^−^^3^
Hall symbol: -P 2ybc	Mo *K*α radiation, λ = 0.71073 Å
*a* = 11.997 (2) Å	θ = 3.0–27.5°
*b* = 7.2959 (15) Å	µ = 0.37 mm^−^^1^
*c* = 9.1453 (18) Å	*T* = 293 K
β = 96.00 (3)°	Block, colorless
*V* = 796.1 (3) Å^3^	0.20 × 0.20 × 0.20 mm

### Data collection

**Table:**

Rigaku SCXmini diffractometer	1827 independent reflections
Radiation source: fine-focus sealed tube	1629 reflections with *I* > 2σ(*I*)
graphite	*R*_int_ = 0.031
CCD_Profile_fitting scans	θ_max_ = 27.5°, θ_min_ = 3.3°
Absorption correction: multi-scan (*CrystalClear*; Rigaku, 2005)	*h* = −15→15
*T*_min_ = 0.955, *T*_max_ = 0.955	*k* = −9→9
7956 measured reflections	*l* = −11→11

### Refinement

**Table:**

Refinement on *F*^2^	Secondary atom site location: difference Fourier map
Least-squares matrix: full	Hydrogen site location: inferred from neighbouring sites
*R*[*F*^2^ > 2σ(*F*^2^)] = 0.034	H-atom parameters constrained
*wR*(*F*^2^) = 0.088	*w* = 1/[σ^2^(*F*_o_^2^) + (0.038*P*)^2^ + 0.4026*P*] where *P* = (*F*_o_^2^ + 2*F*_c_^2^)/3
*S* = 1.11	(Δ/σ)_max_ = 0.001
1827 reflections	Δρ_max_ = 0.26 e Å^−^^3^
109 parameters	Δρ_min_ = −0.36 e Å^−^^3^
0 restraints	Extinction correction: *SHELXL*
Primary atom site location: structure-invariant direct methods	Extinction coefficient: 0

### Special details

**Table:**

Geometry. All e.s.d.'s (except the e.s.d. in the dihedral angle between two l.s. planes) are estimated using the full covariance matrix. The cell e.s.d.'s are taken into account individually in the estimation of e.s.d.'s in distances, angles and torsion angles; correlations between e.s.d.'s in cell parameters are only used when they are defined by crystal symmetry. An approximate (isotropic) treatment of cell e.s.d.'s is used for estimating e.s.d.'s involving l.s. planes.
Refinement. Refinement of *F*^2^ against ALL reflections. The weighted *R*-factor *wR* and goodness of fit *S* are based on *F*^2^, conventional *R*-factors *R* are based on *F*, with *F* set to zero for negative *F*^2^. The threshold expression of *F*^2^ > σ(*F*^2^) is used only for calculating *R*-factors(gt) *etc*. and is not relevant to the choice of reflections for refinement. *R*-factors based on *F*^2^ are statistically about twice as large as those based on *F*, and *R*- factors based on ALL data will be even larger.

### Fractional atomic coordinates and isotropic or equivalent isotropic displacement parameters (Å^2^)

**Table:**

	*x*	*y*	*z*	*U*_iso_*/*U*_eq_	
S1	0.72018 (3)	0.02705 (6)	0.55200 (4)	0.02178 (13)	
C11	0.94220 (13)	−0.0264 (2)	0.49266 (17)	0.0209 (3)	
C12	0.86385 (13)	0.0911 (2)	0.55496 (17)	0.0213 (3)	
C7	0.89809 (14)	0.2508 (2)	0.6239 (2)	0.0291 (4)	
H7	0.8460	0.3258	0.6632	0.035*	
C16	0.91084 (14)	−0.1936 (2)	0.4198 (2)	0.0287 (4)	
H16	0.8360	−0.2294	0.4101	0.034*	
C8	1.01178 (15)	0.3021 (3)	0.6358 (2)	0.0347 (4)	
H8	1.0342	0.4115	0.6822	0.042*	
N1	0.39695 (11)	0.03920 (19)	0.90988 (15)	0.0230 (3)	
H1A	0.3451	0.1206	0.8730	0.028*	
H1B	0.3762	−0.0722	0.8746	0.028*	
C5	0.40092 (15)	0.0374 (3)	1.07288 (19)	0.0292 (4)	
H5A	0.3293	−0.0036	1.1011	0.035*	
H5B	0.4143	0.1607	1.1104	0.035*	
C1	0.50731 (14)	0.0883 (3)	0.86030 (18)	0.0270 (4)	
H1C	0.5256	0.2139	0.8882	0.032*	
H1D	0.5027	0.0802	0.7540	0.032*	
O1	0.67590 (11)	0.0023 (2)	0.39986 (15)	0.0384 (3)	
O3	0.66605 (10)	0.17560 (17)	0.62410 (15)	0.0334 (3)	
O2	0.72065 (10)	−0.14134 (17)	0.63779 (14)	0.0312 (3)	

### Atomic displacement parameters (Å^2^)

**Table:**

	*U*^11^	*U*^22^	*U*^33^	*U*^12^	*U*^13^	*U*^23^
S1	0.0183 (2)	0.0221 (2)	0.0249 (2)	−0.00043 (14)	0.00176 (15)	0.00085 (15)
C11	0.0206 (8)	0.0213 (8)	0.0205 (8)	−0.0020 (6)	0.0011 (6)	−0.0019 (6)
C12	0.0196 (7)	0.0220 (8)	0.0221 (8)	−0.0016 (6)	0.0015 (6)	0.0005 (6)
C7	0.0253 (8)	0.0261 (9)	0.0362 (10)	0.0007 (7)	0.0047 (7)	−0.0093 (7)
C16	0.0225 (8)	0.0279 (9)	0.0357 (10)	−0.0072 (7)	0.0024 (7)	−0.0091 (8)
C8	0.0309 (9)	0.0290 (9)	0.0440 (11)	−0.0080 (7)	0.0032 (8)	−0.0176 (8)
N1	0.0229 (7)	0.0229 (7)	0.0221 (7)	0.0017 (5)	−0.0022 (5)	0.0011 (5)
C5	0.0282 (9)	0.0363 (10)	0.0235 (8)	0.0051 (7)	0.0047 (7)	0.0003 (7)
C1	0.0275 (8)	0.0321 (9)	0.0212 (8)	−0.0028 (7)	0.0012 (6)	0.0070 (7)
O1	0.0290 (7)	0.0556 (9)	0.0284 (7)	−0.0015 (6)	−0.0070 (5)	−0.0012 (6)
O3	0.0273 (6)	0.0267 (7)	0.0478 (8)	0.0040 (5)	0.0120 (6)	−0.0017 (6)
O2	0.0324 (7)	0.0230 (6)	0.0386 (7)	−0.0042 (5)	0.0051 (5)	0.0053 (5)

### Geometric parameters (Å, °)

**Table:**

S1—O1	1.4477 (14)	C8—C16^i^	1.359 (3)
S1—O3	1.4562 (13)	C8—H8	0.9300
S1—O2	1.4574 (13)	N1—C5	1.486 (2)
S1—C12	1.7834 (16)	N1—C1	1.487 (2)
C11—C16	1.422 (2)	N1—H1A	0.9000
C11—C11^i^	1.432 (3)	N1—H1B	0.9000
C11—C12	1.434 (2)	C5—C1^ii^	1.512 (2)
C12—C7	1.367 (2)	C5—H5A	0.9700
C7—C8	1.408 (2)	C5—H5B	0.9700
C7—H7	0.9300	C1—C5^ii^	1.512 (2)
C16—C8^i^	1.359 (3)	C1—H1C	0.9700
C16—H16	0.9300	C1—H1D	0.9700
			
O1—S1—O3	113.07 (8)	C7—C8—H8	119.6
O1—S1—O2	113.12 (8)	C5—N1—C1	111.82 (13)
O3—S1—O2	111.13 (8)	C5—N1—H1A	109.3
O1—S1—C12	107.70 (8)	C1—N1—H1A	109.3
O3—S1—C12	105.90 (8)	C5—N1—H1B	109.3
O2—S1—C12	105.28 (8)	C1—N1—H1B	109.3
C16—C11—C11^i^	118.75 (18)	H1A—N1—H1B	107.9
C16—C11—C12	123.17 (15)	N1—C5—C1^ii^	110.87 (14)
C11^i^—C11—C12	118.07 (18)	N1—C5—H5A	109.5
C7—C12—C11	121.01 (15)	C1^ii^—C5—H5A	109.5
C7—C12—S1	118.27 (13)	N1—C5—H5B	109.5
C11—C12—S1	120.66 (12)	C1^ii^—C5—H5B	109.5
C12—C7—C8	120.27 (16)	H5A—C5—H5B	108.1
C12—C7—H7	119.9	N1—C1—C5^ii^	111.37 (14)
C8—C7—H7	119.9	N1—C1—H1C	109.4
C8^i^—C16—C11	121.18 (16)	C5^ii^—C1—H1C	109.4
C8^i^—C16—H16	119.4	N1—C1—H1D	109.4
C11—C16—H16	119.4	C5^ii^—C1—H1D	109.4
C16^i^—C8—C7	120.71 (16)	H1C—C1—H1D	108.0
C16^i^—C8—H8	119.6		

Symmetry codes: (i) −*x*+2, −*y*, −*z*+1; (ii) −*x*+1, −*y*, −*z*+2.

### Hydrogen-bond geometry (Å, °)

**Table:**

*D*—H···*A*	*D*—H	H···*A*	*D*···*A*	*D*—H···*A*
N1—H1A···O2^iii^	0.90	1.91	2.7357 (19)	153.
N1—H1B···O3^iv^	0.90	1.91	2.7670 (19)	159.

Symmetry codes: (iii) −*x*+1, *y*+1/2, −*z*+3/2; (iv) −*x*+1, *y*−1/2, −*z*+3/2.
